# Advances in research on factors affecting chimeric antigen receptor T‐cell efficacy

**DOI:** 10.1002/cam4.7375

**Published:** 2024-06-12

**Authors:** Delian Zhou, Xiaojian Zhu, Yi Xiao

**Affiliations:** ^1^ Department of Hematology, Tongji Hospital, Tongji Medical College Huazhong University of Science and Technology Wuhan Hubei China

**Keywords:** chimeric antigen receptor T‐cell, lymphodepletion chemotherapy, therapeutic effect

## Abstract

Chimeric antigen receptor T‐cell (CAR‐T) therapy is becoming an effective technique for the treatment of patients with relapsed/refractory hematologic malignancies. After analyzing patients with tumor progression and sustained remission after CAR‐T cell therapy, many factors were found to be associated with the efficacy of CAR‐T therapy. This paper reviews the factors affecting the effect of CAR‐T such as tumor characteristics, tumor microenvironment and immune function of patients, CAR‐T cell structure, construction method and in vivo expansion values, lymphodepletion chemotherapy, and previous treatment, and provides a preliminary outlook on the corresponding therapeutic strategies.

## 1 INTRODUCTION

Chimeric antigen receptor T‐cell (CAR‐T) therapy is an innovative immunotherapeutic approach that genetically modifies T cells to express specific antigen receptors, enabling them to recognize antigens present on the surface of tumor cells. This therapy has demonstrated remarkable efficacy in treating relapsed/refractory acute lymphoblastic leukemia (ALL),^1^, ^2^, ^3^ chronic lymphoblastic leukemia (CLL), non‐Hodgkin's lymphoma (NHL),^4^, ^5^, ^6^ and multiple myeloma (MM).^7^, ^8^, ^9^, ^10^ Notably, CD19 CAR‐T cells have achieved complete remission (CR) rates ranging from 62% to 86% in B‐cell ALL (B‐ALL) patients who are negative for micro‐residual disease (MRD).^11^ Furthermore, CAR‐T cells targeting B‐cell maturation antigen (BCMA) have shown high efficacy in the treatment of relapsed/refractory MM (R/R MM), with overall response rates (ORR) ranging from 73% to 98%.^12^, ^13^, ^14^ CAR‐T cell therapy has emerged as a crucial component in the treatment of hematological malignancies and holds promising prospects for further development. However, some patients exhibit poor efficacy, with a relapse rate of up to 60% after BCMA CAR‐T therapy, and the durability of remission after relapse salvage therapy is unsatisfactory.^15^ Another study also concluded that only 8% to 39% of MM patients receiving BCMA CAR‐T therapy achieved sustained very good partial response (VGPR) or CR/stringent complete response (sCR), with a median progression‐free survival (PFS) of only 8.8 months.^16^ Cappell et al. reviewed the long‐term outcomes of CAR T cell therapy, highlighting the therapeutic efficacy and durability of CD19‐ and BCMA‐targeted CAR T cells across various patient populations. Their study indicated that, while CD19‐targeted CAR T cells exhibited notable long‐term remission in patients with B‐cell malignancies, the remission duration of BCMA‐targeted CAR T cells in MM patients was relatively short.^17^ Therefore, comprehending the factors influencing CAR‐T cell efficacy is crucial for augmenting CAR‐T cell cytotoxicity and ameliorating patient outcomes.

**FIGURE 1:**
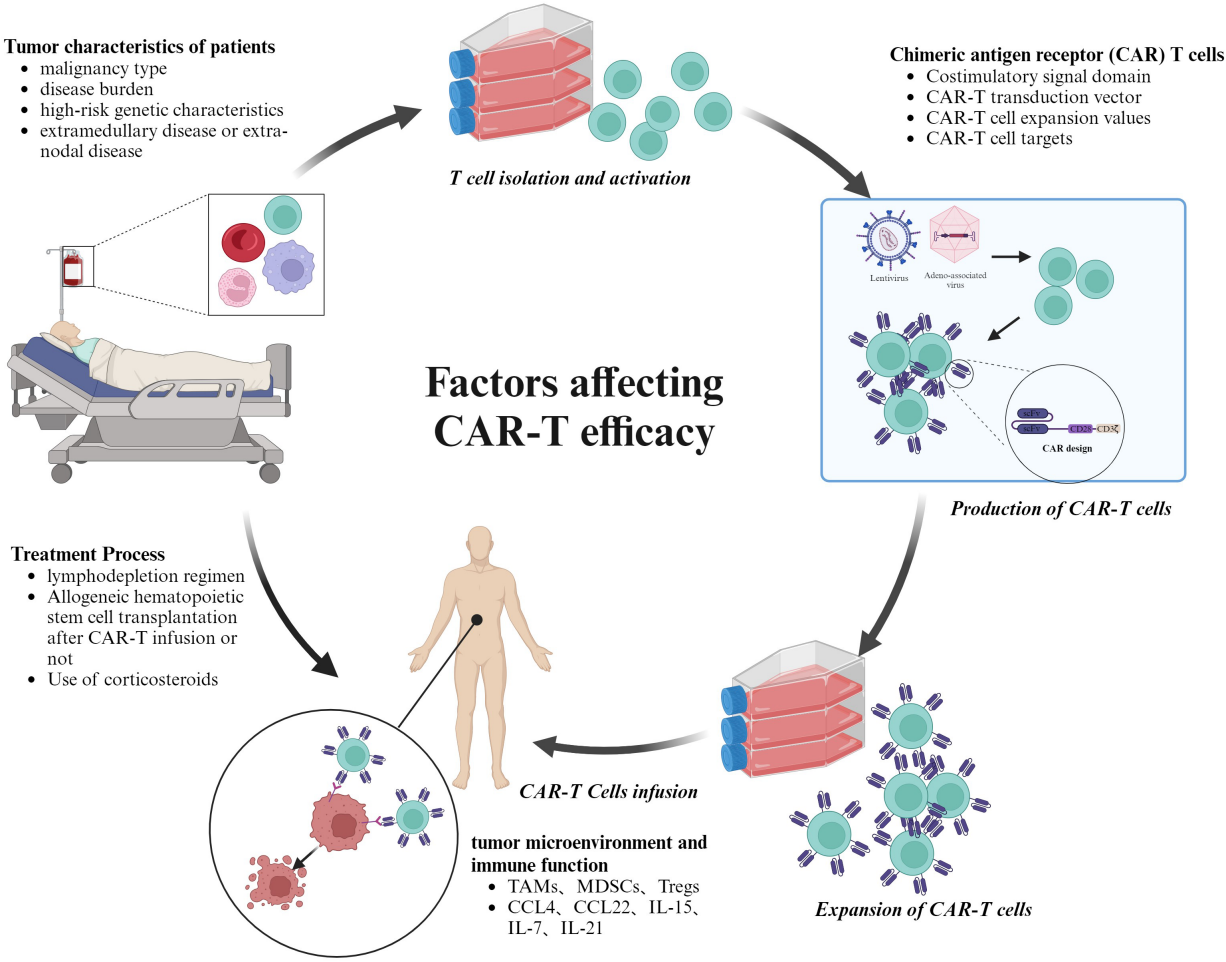
The construction process of CAR‐T cells and the factors that impact their efficacy.

The standard procedure for CAR‐T therapy involves several key steps^18^: patient screening, collection of peripheral blood mononuclear cells (PBMCs), optional bridging therapy, lymphodepletion chemotherapy, CAR‐T cell infusion, post‐transfusion monitoring, adverse reaction management, and long‐term post‐transfusion follow‐up. Various factors influencing long‐term remission were discussed by Cappell, including the depth of the initial response, peak levels of circulating CAR‐T cells, tumor type, tumor burden, and location, as well as the role of lymphodepleting chemotherapy.^17^ Additionally, researchers addressed the impact of the tumor microenvironment,^19^ patient selection, and management during CAR‐T therapy,^20^ and summarized optimization strategies to overcome these challenges. At the same time, potential solutions have been proposed for issues such as primary and acquired resistance, poor in vivo persistence of CAR‐T cells, high treatment costs, and serious side effects, including cytokine release syndrome (CRS) and immune effector cell‐associated neurotoxicity syndrome (ICANS), during the treatment process.^21^ These strategies include constructing universal CAR‐T cells, utilizing dual‐target/multi‐target CAR‐T cells, optimizing the CAR‐T manufacturing process, adopting combination therapies, and implementing salvage therapy post‐CAR‐T treatment.^22^ These studies provide important references for further exploring the factors affecting CAR‐T cell efficacy. Building on this foundation, we systematically summarize various aspects that may affect the efficacy of CAR‐T cell therapy, encompassing patient tumor characteristics, CAR‐T cells, tumor microenvironment, immune function, treatment processes, and other factors. We supplement the aforementioned research with overlooked influencing factors and integrate the latest research advancements to promote the broader application of CAR‐T cell therapy in cancer treatment (Figure 1).

## 2 TUMOR CHARACTERISTICS OF PATIENTS

### 2.1 Malignancy type

Malignancy type predicts the duration of remission. Patients with B‐cell lymphoma^23^, ^24^, ^25^, ^26^ have a lower likelihood of achieving CR compared to those with B‐ALL^27^, ^28^, ^29^, ^30^, ^31^, ^32^ or MM^14^, ^33^, ^34^; however, patients with B‐cell lymphoma have a higher chance of achieving long‐lasting remission compared to other types of disease. In addition, due to the presence of tumor‐associated antigens on both normal hematopoietic stem cells and mature blood cells in most AML patients, targeting the tumor can lead to simultaneous attacks on normal tissues. This off‐target toxicity limits the application of CAR‐T therapy in AML. Currently, common targeted antigens include CD33, CD123, CCL1, among others,^35^ but the efficacy remains poor, with a less than 20% 5‐year survival rate. In summary, the type of malignancy significantly influences treatment outcomes. Finding appropriate targets and more effective treatment methods are crucial for improving patient survival rates.

### 2.2 Disease burden

The disease burden can predict patient prognosis to varying degrees. In all hematological malignancies, individuals with a higher initial tumor burden are generally less likely to achieve and sustain a significant treatment response compared to those with a lower tumor burden.^14^, ^36^, ^37^, ^38^ In 2018, researchers conducted a phase 1 trial involving 53 adult patients who had relapsed B‐ALL. These patients received an infusion of CAR‐T cells called 19‐28z. The study showed that 83% of patients achieved CR, with a median overall survival (OS) of 12.9 months. Among patients with a low disease burden (<5% bone marrow blasts), the median OS was extended to 20.1 months. Additionally, these patients experienced a lower incidence of CRS and neurotoxicity. It is worth noting that patients with a low disease burden exhibited longer long‐term survival compared to those in the high disease burden group (≥5% bone marrow blasts or extramedullary disease).^28^

At the 2020 American Society of Hematology (ASH) annual meeting, Schultz et al. reported the real‐world clinical results for pediatric patients with relapsed/refractory B‐ALL (R/R B‐ALL) who treated with commercialized Tisacel: 185 patients from 15 institutions were infused with Tisacel, the OS and event‐free survival (EFS) rates at 6 and 12 months, respectively, and a lower incidence of CRS and ICANS. A comparative analysis of the results of cohorts of patients with different disease burdens showed that patients with high disease burden before CAR infusion had lower morphological CR rates at day 28 (74% vs. 98% and 96%), lower 6‐month EFS (50% vs. 86% and 75%) and OS (75% vs. 94% and 98%), and lower 1‐year EFS (34% vs. 69% and 72%) and OS (58% vs. 85% and 95%) compared to patients with low disease burden and those who did not achieve disease detection at the last assessment.^39^ Therefore, the disease burden on patients before treatment is a useful predictor of remission and survival rates.

Moreover, lactate dehydrogenase (LDH) levels are associated with disease burden, and glycolysis in hematological malignant tumor cells is higher than that in normal tissue cells. Therefore, the enzymes involved in glycolysis, namely LDH, are also elevated. Accelerated metabolic conversion rates, tumor cell necrosis, and changes in cell membrane permeability lead to the release of this enzyme from tumor cells into the blood, resulting in increased serum LDH levels. Conversely, when tumor tissues infiltrate or invade normal tissues, the cellular structures are compromised, leading to the release of LDH from normal tissues into the blood, further elevating blood LDH levels. Notably, the International Prognostic Index score for lymphoma incorporates LDH level as a factor. Thus, elevated LDH are correlated with increased tumor burden within the body and are indicative of a poor prognosis in lymphoma. Related studies have shown that serum LDH concentration and platelet count have a significant impact on the prognosis of patients with hematologic malignancies,^40^ and these factors may impact disease burden.

### 2.3 High‐risk genetic characteristics

Cytogenetic abnormalities in CAR‐T therapy have significant clinical implications. Patients with high‐risk cytogenetics, such as TP53 mutations,^41^ Ph+, Ph‐like, KMT2A‐rearranged,^42^ hypodiploid and the presence of a t(17;19)(q22;p13) leads to the fusion of TCF3 and HLF genes.^43^ This research emphasizes the clinical significance of cytogenetic abnormalities in CAR‐T therapy. Notably, the TP53 gene is a clear indicator of poor prognosis. This tumor suppressor gene is located on chromosome 17q13.1 and is 19,144 nucleotides long, encompassing 11 exons. It encodes the P53 protein, which consists of 393 amino acids and functions as a crucial tumor suppressor. Importantly, the DNA‐binding region (DBD region) is vital for the functionality of the TP53 gene. Mutations in TP53 have garnered increasing attention in determining the prognosis of clinical hematological diseases. Specifically, in a clinical trial evaluating CD19 CAR‐T cells for R/R B‐ALL, Zhang et al. concluded that patients with TP53 mutations exhibited significantly lower OS (51.9% vs 89.0%) and leukemia‐free survival (LFS) (42.4% vs. 82.6%) at 6 months compared to those without TP53 mutations, albeit with no significant difference in CR rates.^44^ Another study also concluded that TP53 abnormalities serve as prognostic markers for CD19 CAR‐T therapy, with a 1‐year OS rate of 44% for TP53 abnormal DLBCL patients compared to 76% for wild‐type.^45^ Furthermore, Ph+, caused by a chromosomal translocation known as t(9;22)(q34;q11), resulting in a BCR/ABL fusion gene, is predominantly observed in chronic and acute myeloid leukemia and ALL. CAR‐T therapy for R/R Ph+ ALL shows some efficacy in children, but the treatment outcomes in adults are inferior to those in children. Despite a relatively high remission rate, the relapse rate ranges from 20% to 70%, and the median LFS is only 5.3–10.6 months,^28^, ^46^ possibly associated with the complex molecular biology background. Patients with this genetic abnormality also demonstrate a poor prognosis.

### 2.4 Extramedullary disease or extra‐nodal disease

The presence of extranodal B‐cell lymphoma,^47^ extramedullary B‐ALL,^40^, ^41^, ^42^, ^43^, ^44^, ^45^, ^46^, ^47^, ^48^ or extramedullary MM^33^, ^49^ is another factor that can impact the prognosis of different malignancies, all of which portend a poorer prognosis. In patients diagnosed with diffuse large B‐cell lymphoma (DLBCL), specific primary sites can be identified, such as the central nervous system (CNS), testicles, and mediastinum. Notably, the prognosis for these lymphomas, when located outside the primary lymph nodes, is significantly poor.^47^ The OS rate and replase‐free survival (RFS) in leukemia patients was found to be lower in those with CNS involvement compared to those without CNS involvement.^48^ Therefore, special attention must be paid to the prognosis of patients with extranodal and extramedullary lesions, and more aggressive treatment strategies should be adopted. Additionally, early detection and management of high‐risk area involvement, such as the central nervous system, are crucial for improving overall patient prognosis.

### 2.5 Others

Platelet counts prior to lymphodepletion chemotherapy correlate with treatment efficacy. Specifically, higher platelet counts have been linked to prolonged PFS in patients following CAR‐T treatment^40^ and can thus serve as a predictor of CAR‐T efficacy. Pediatric and young adult patients experience a longer median EFS compared to adults, indicating there is a correlation between the effectiveness of CAR‐T cell therapy and age. This may be explained by the fact that older CAR‐T cells have a shorter lifespan, are less efficient at transduction, are more cytotoxic, and have a higher relapse. Furthermore, the higher the number of previous lines of treatment, the worse the patient's prognosis.

## 3 TUMOR MICROENVIRONMENT AND IMMUNE FUNCTION

The immunosuppressive microenvironment contains a large number of cells and cytokines, including tumor‐associated macrophages^50^ (especially M2 macrophages), regulatory T cells (Tregs), endothelial cells, myeloid‐derived suppressor cells, osteoblasts and fibroblasts. The tumor microenvironment is crucial in tumor development, including the following aspects: (i) It exerts significant regulatory effects on tumor gene mutations and expressions, directly or indirectly impacting tumor growth and metastasis. (ii) Immunosuppressive cell and immunosuppressive factors in the microenvironment promote immune escape and tumor progression. (iii) Abundant cytokines and growth factors such as VEGF, FGF, and CXCL12 in the tumor microenvironment promote angiogenesis, providing tumors with more nutrients. (iv) The matrix facilitates the invasion and migration of tumor cells.^51^, ^52^ Research indicates that the immunosuppressive microenvironment that restricts the transport of CAR‐T cells to tumor cells, thereby affecting the killing effect of CAR‐T cells on tumor cells.^53^ In conclusion, understanding and addressing the immunosuppressive microenvironment are essential for optimizing the efficacy of CAR‐T cell therapy in cancer treatment. Further research and clinical trials are needed to develop effective strategies that can overcome these obstacles and improve the outcomes of CAR‐T cell therapy for cancer patients.

### 3.1 Immune cells

The different stages of T cell differentiation include naive T cells, stem cell central memory T cells (TSCM), central memory T lymphocytes (TCM), effector memory T lymphocytes (Tem), and effector T cells. The therapeutic effectiveness and durability of CAR‐T cells are closely correlated with their differentiation status. The optimal prognostic indicator ought to be evaluated in T cells immediately procured from the peripheral blood of patients, including T cell activation levels and the proportion of T cell subsets,^53^, ^54^ to ascertain whether expansion is requisite or if more efficacious methods are needed to augment the specific expansion of primary T cells. Importantly, according to several studies, there is a correlation between the immune background preceding treatment and survival outcomes. Notably, among them, activated CD8+ T cells were found to be associated with the ORR rate, compared to other CD8+ T cells, such as inactivated CD8+ T cells (no checkpoint expression) or depleted T cells.^53^ By evaluating the different subpopulations of T cells in the tumor environment prior to CAR‐T cell infusion, it has been noted that patients with higher densities of helper T cells demonstrated a greater likelihood of achieving CR and enhanced survival rates; conversely, those with elevated densities of Tregs were less likely to attain CR.^54^ Therefore, detailed analysis of patient T cell subsets before treatment can provide important references for developing personalized CAR‐T cell therapy plans. Additionally, regulatory strategies targeting specific T cell subsets may further enhance the efficacy of CAR‐T cell therapy and improve long‐term survival rates of patients.

In addition, macrophages have both pro‐ and anti‐tumorigenic roles, with the pro‐tumorigenic cell population being termed tumor‐associated macrophages (TAM). Metabolites enriched in the tumor microenvironment control the polarization of macrophages, leading to a pro‐tumorigenic phenotype. These metabolites also enhance the inhibitory activity of Tregs. The combined activation of both TAM and Tregs exerts a suppressive function on CD8+ T cells, leading to adverse clinical outcomes. The main mechanism involves NO produced by TAM impairing cytotoxic T lymphocyte (CTL) activation through inhibition of the Janus kinase‐3 (JAK3) and transcription activator of transcription‐5 (STAT5) signaling pathways; TAM depletion of nutrients in the microenvironment reduces CTL proliferation; CTL, upon sensing glucocorticoids produced by TAM, inhibit the secretion of effector cytokines and induce the expression of inhibitory receptors and immunosuppressive factor (IL‐10); TAM and Treg interaction forms a positive feedback loop that impairs anti‐tumor immunity and leads to poor clinical outcomes.^55^ Clarifying the role of TAM in tumor cells and other immune cells enhances the understanding of the immunosuppressive mechanism during CAR‐T therapy and offers new therapeutic targets for subsequent treatment. Dendritic cells, as key antigen‐presenting cells, regulate anti‐tumor immune responses by integrating and delivering information to T cells.^56^ Neutrophils have both pro‐ and anti‐tumor functions. Neutrophils with an anti‐tumor phenotype can clear mutated tumors resulting from antigen loss^57^ during CAR‐T therapy. Additionally, studies have concluded that high levels of neutrophils are positively correlated with the success of tumor immunotherapy. It was found that the mechanism of tumor suppression involves an increase in interferon‐responsive transcription factor (IRF1), BATF3‐dependent dendritic cells, IL‐12, and IFN‐γ in neutrophils, further emphasizing the key role of neutrophils in this therapy.^58^ Importantly, this suggests that the patinets' immune function is a critical component that cannot be overlooked.

### 3.2 Cytokines

Interleukin‐15 (IL‐15) is a crucial pro‐inflammatory factor with multifaceted functions, such as T cell regulation, tissue repair, B cell homing, inflammation modulation, and NK cell activation, among other functions.^59^ IL‐15 exerts its biological functions by interacting with its corresponding receptor (IL‐15R), comprised of three subunits, αβγ. Notably, only the α chain is highly specific. Upon secretion, IL‐15 predominantly binds to the high affinity IL‐15Rα on the cell membrane of adjacent antigen‐presenting cells, forming the IL‐15/IL‐15Rα complex. This complex is subsequently transpresented to the IL‐2/15Rβγ heterodimer, thereby activating the downstream JAK/STAT pathway, MAPK pathway, and PI3K/AKT pathway.^60^ Consequently, this interaction influences gene transcription and modulates the immune activation, proliferation, and apoptotic responses of effector cells. Importantly, IL‐15 is important in both innate and adaptive immunity as a potential immunostimulatory molecule.^61^ Moreover, it possesses additional benefits, such as the absence of Tregs activation, diminished activation‐induced cell death, and reduced cytotoxicity.^62^ Based on these results, we speculate that supplementing IL‐15 during CAR‐T therapy could enhance the survival of CAR‐T cells.

In a clinical trial (NCT01865617), CD19 CAR‐T cells were evaluated for the treatment of B‐cell malignancies, the authors posited that IL‐15 enhances T‐cell proliferation and survival.^63^ Furthermore, they suggested that the challenges linked to suboptimal kinetics following CAR‐T cell infusion might be mitigated through supplementation with IL‐15. NKTR‐255 is an IL‐15 receptor agonist, in vitro, it induces growth and accumulation of human CD19 CAR‐T cells in a dose‐dependent manner. Furthermore, studies in immunodeficient mice with lymphoma have shown an improved effectiveness of human CD19 CAR‐T cells when supplemented with NKTR‐255, enhancing their ability to combat tumors. Compared to those of mice treated with CAR‐T cells individually, CAR‐T cell numbers in the blood and bone marrow of mice treated with NKTR‐255 and CAR‐T cells were significantly increased and remained after tumor clearance.^63^ Thus, it is hypothesized that serum IL‐15 levels prior to CAR‐T infusion may predict the kinetics of CAR‐T cell expansion and, consequently, extend to the prediction of clinical effects.

Severe CRS and ICANS are major challenges in CAR‐T therapy, and the abnormal increase of cytokines such as interleukin‐6 (IL‐6) is a characteristic manifestation of CRS.^64^ A report at ASH 2023 demonstrated the safety and efficacy of using a small hairpin RNA (shRNA) element to silence the IL‐6 gene in CD19 CAR‐T (ssCAR‐T) cells for the treatment of R/R B‐ALL. Compared with classical CD19 CAR‐T (cCAR‐T), the ssCAR‐T group had a higher 3‐month PFS (82.3% vs 66.9%), but no significant difference in 3‐year PFS or OS. ssCAR‐T patients had a lower probability of CRS (68.09% vs 85.0%) and ICANS (4.26% vs 15%), and the incidence of other adverse events was also significantly reduced.^65^ This suggests that silencing IL‐6 facilitates the functionality of CD19 CAR‐T cells and reduces toxic side effects, making it a promising therapeutic approach. Further, the lower the IL‐6 level, the easier it is to achieve a durable response. In addition, Tocilizumab, a widely used IL‐6R monoclonal antibody in clinical practice, also significantly reduced the incidence of CRS.^66^ Moreover, it did not affect the expansion of CAR‐T cells when compared to the non‐draglumab group, while reducing adverse effects.

Transforming growth factor β (TGF‐β), as a negative regulatory factor, is primarily secreted by immunosuppressive cells or tumor cells in the tumor microenvironment.^67^ It binds to TGF‐βI receptors (TGF‐βRI or ALK5) and TGF‐βII receptors (TGF‐βRII), leading to phosphorylation of the intracellular domain of TGF‐βRI and subsequent activation of downstream SMAD proteins to exert its function.^68^ CD26, a multifunctional protein present on the surface of T cells, promotes T cell proliferation. However, in the tumor microenvironment, TGF‐β‐1 inhibits the expression of CD26, thereby reducing T cell activity. Consequently, in CAR‐T therapy for malignant hematological tumors, increased levels of TGF‐β may weaken the effector function of CAR‐T cells.^69^ Studies have shown that knocking out the endogenous TGF‐β receptor II (TGFBR2) in CAR T cells using CRISPR/Cas9 technology can reduce the induction of Treg transformation and prevent exhaustion of CAR T cells.^70^ Thus, the increase of TGF‐β in the tumor microenvironment during CAR‐T therapy may diminish the cytotoxic effects of CAR‐T cells, limiting their ability to recognize and eliminate tumor cells, thereby affecting treatment outcomes. Therefore, targeting the TGF‐β signaling pathway is crucial for enhancing the efficacy of CAR‐T cell therapy.

Importantly, Interferon γ (IFN‐γ), C‐C motif chemokine ligand 2 (CCL2) and CXC chemokineligand‐10 (CXCL10) are correlated with Grade 3–4 ICANS or CRS.^71^ Therefore, monitoring cytokines in the tumor microenvironment and intervening promptly can enhance the survival and efficacy of CAR‐T cells.

## 4 CAR‐T CELLS

The steps in CAR‐T cell therapy include the extraction of T cells from the patients' peripheral blood,^72^ T cell activation, gene transduction using viral or non‐viral vectors, CAR‐T cell expansion in vitro, cell therapy product treatment monitoring, cryopreservation, and return to the patient. Each step may ultimately affect the CAR‐T efficacy throughout the manufacturing process.

### 4.1 Costimulatory signal domain

CAR T cell structure includes extracellular domain for antigen recognition and binding, transmembrane domain, costimulatory signal domain, and intracellular signal transduction domain (CD3ζ chain).^73^, ^74^ The antigen recognition‐binding region is usually a single‐chain variable fragment (scFv). This fragment is primarily made up of the antibody's variable light chain, variable heavy chain, and a linker region in between. The transmembrane region serves as an anchor for CAR molecules on the cell membrane, contributing to the stability of CAR molecular expression. The costimulatory signal domain is tasked with delivering a second activation signal to T cells, and in second‐generation CAR T cells, this domain is either 4‐1BB or CD28.^75^ The intracellular signal transduction domain is the CD3ζ chain, which is widely utilized as the standard molecular signaling domain in CARs.

Some studies on the clinical outcomes of patients after CAR‐T cell infusion have identified the costimulatory signal domain to be one of the factors affecting clinical efficacy. Anagnostou et al. reported outcome data in adult and pediatric patients with R/R B‐ALL treated with anti‐CD19 CAR‐T cells. In the final analysis, a total of 953 patients were included, and analysis of CD19 CAR T‐cell construct types showed that patients treated with the 4‐1BB costimulatory domain had a greater proportion of undetectable MRDs than did those treated with CD28.^76^ In addition, CAR‐T cells incorporating a CD28 costimulatory domain proliferated at a fast rate and high level but for a short duration, whereas CAR‐T cells incorporating a 4‐1BB costimulatory domain proliferated relatively slowly but for a long duration.^77^, ^78^, ^79^ Therefore, the CD28 costimulatory domain showed less durable in vivo persistence compared to the 4‐1BB costimulatory domain in CAR‐T cells.^80^ In summary, CAR‐T cells with different costimulatory domains exhibit varying levels of proliferation and persistence. Research has identified costimulatory domains as critical factors in CAR‐T cell dysfunction, with different costimulatory domains leading to distinct dysfunction mechanisms. Specifically, the CD28 costimulatory domain initiates the exhaustion program in CAR‐T cells, whereas the 4‐1BB costimulatory domain induces a novel cellular state in T cells via the activation of the transcription factor FOXO3, ultimately leading to T cell dysfunction.^81^ To enhance the efficacy of CAR‐T therapy, implementing targeted interventions to address these specific limitations is essential.

### 4.2 CAR‐T transduction vector

As the core part of CAR‐T production and preparation, the gene delivery vector directly affects the ultimate therapeutic efficacy of CAR‐T products in clinical applications. Vectors used for CAR‐T transduction include lentiviral vectors (LV), γ‐retroviral vectors, adenovirus‐associated vectors (AAV), synthetic polymer nanocarriers (NC), and lipid nanoparticles (LNP). Lentiviruses have a wide range of target cells, including activated T lymphocytes, and are commonly used for efficient in vitro transduction in CAR therapy, and CAR therapy depends primarily on in vitro transduction of lentiviral or γ‐retroviral vectors. There have been numerous clinical trials conducted or currently ongoing, mostly using lentiviruses to treat immune and hematologic diseases and cancers.^81^, ^82^, ^83^ AAV are distinct from LV in many ways, it comprises a non‐cytosolic protein capsid that houses a single‐stranded DNA genome; therefore, its genetic modifications are usually transient,^84^ and after interacting with the target receptor of the cell, clathrins mediate endocytosis and intracellular transport, and AAV enters the nucleus and releases single‐stranded genes used for transcription.^85^ Nonviral vectors exhibit less order than viral vector particles. Contrary to the intricate mechanisms of viral vectors, the processes of cell entry, transport, and transgene delivery mediated by nonviral vectors are dictated by the physicochemical properties of particles and additional payloads.^86^ NC is a complex of negatively charged nucleic acids and positively charged polymers. Upon cell uptake, the nucleic acids of NC escape from endosomes and enter the cells. Moreover, in LNP, the core of the lipid particles encapsulates mRNA through electrostatic interactions.^87^ Once taken up by host cells and successfully escaping the endosome, the mRNA remains accessible for translation until its eventual degradation. The transfection method based on LNP has been shown to enhance the activity of T cells in vitro, prolong the expression time of CAR on T cells, and significantly improve the long‐term therapeutic efficacy of CAR‐T cells.^88^ Researchers have also developed an engineered LNP platform capable of simultaneously delivering mRNA encoding CAR and siRNA targeting PD‐1 mRNA to T cells. By targeting both tumor cells and immune inhibitory signals, this platform transiently disrupts the PD‐1 signal, allowing the restoration of normal T cell function in patients.^89^ Through continuous optimization and improvement, the engineered LNP platform holds promise as an effective and safe gene delivery system, bringing forth additional possibilities for clinical therapy.

The main vectors used in the clinic are lentiviral and retroviral transduction.^90^ However, in recent years, γ‐retroviral transduction has been gradually replaced by lentiviral transduction in clinical practice due to the risk of causing insertional oncogenesis, inability to infect non‐dividing cells, and low viral titers. γ‐retroviruses emonstrate a preference for integrating near transcription start sites and CpG islands,^91^ which may lead to cellular oncogenesis. In contrast to retroviruses, lentiviruses preferentially integrate into introns of genes, thereby exhibiting a relatively low oncogenic risk. Moreover, lentiviruses can carry a larger exogenous gene fragment compared to γ‐retroviruses and possess the capacity to infect both dividing and nondividing cells, coupled with reduced immunogenicity. Gils Roex et al. conducted a meta‐analysis utilizing PFS data from 551 patients, it was utilized to determine the median PFS for individuals treated with BCMA CAR‐T cells, which was found to be 12.2 months (11.4–17.4). Notably, In comparison to patients treated with retroviral constructs, the study demonstrated that patients undergoing lentiviral transduction of CAR‐T cells experienced a significantly prolonged PFS (12.8 months [11.4–19.9] vs. 4.3 months [3.0–15.0]).^80^

Although lentiviral vectors have many advantages over other vectors, they also have certain limitations. The primary source of lentivirus is the human immunodeficiency virus, and users harbor concerns regarding its biosafety. Research into safety and the optimization of the lentivirus preparation process remain areas that warrant continuous attention and exploration.

### 4.3 Source of CAR‐T cells

Compared to individuals received with allogeneic CAR‐T cells, patients who receive autologous CAR‐T cells show a greater improvement in CR rates and a higher incidence of ICANS.^76^ However, another study conducted on R/R T‐cell malignancies involved the use of autologous or allogeneic CD7 CAR‐T therapy, involving a total of 10 patients. The results showed that the CR rate for autologous cell therapy was 40%, while for allogeneic therapy it was 80%, indicating a higher efficacy of allogeneic CAR‐T cells. The study also found no significant relationship between the peak expansion of CAR‐T cells and their source. Single‐cell transcriptome sequencing revealed that CD7 deletion occurred in the PBMCs of patients undergoing allogeneic CAR‐T therapy. This deletion may lead to a reduction in the proportion of Tregs in the immune microenvironment and alleviate CAR‐T cell exhaustion resulting from chronic antigen stimulation.^81^ These results suggest that one possible mechanism for the long‐term survival of allogeneic CAR‐T cells could be related to CD7 deletion. The study highlighted the advantages of allogeneic CAR‐T cells in treatment efficacy and proposed that CD7 deletion may play a key role in their long‐term survival. In the treatment of R/R B‐cell precursor acute lymphoblastic leukemia (R/R BCP‐ALL), allogeneic CD19 CAR‐T cells have demonstrated significant efficacy when compared to autologous CD19 CAR‐T cells, while not increasing toxicity. Specifically, they induced 100% MRD‐negative CR in the bone marrow (BM) and 83% CR in extramedullary sites.^92^ In clinical studies utilizing allogeneic BCMA CAR‐T (ALLO‐715) therapy in patients with MM, it was found that 55.8% of patients responded to the treatment, with 34.9% achieving a very good partial response or better (VGPR+). The median duration of response was 8.3 months. Both the incidence of CRS and the incidence of ICANS were 55.8% and 14%.^7^ These results indicate that allogeneic BCMA CAR‐T therapy is effective for the treatment of R/R MM patients, with manageable side effects. In summary, more experimental data are needed to support the exact effects. What's more, CAR‐T products from different manufacturers and institutions vary greatly, which may affect the effectiveness of CAR‐T cell treatment.

### 4.4 CAR‐T cell expansion values

Peak CAR T‐cell amplification values serve as a key indicator for short‐term effects and toxic responses,^93^, ^94^, ^95^ achieving CR with negative MRD following CAR‐T infusion is significantly correlated with higher peak CAR T‐cell amplification compared with a state of no response (NR) or positive MRD. In addition, there was a positive correlation between higher peak CAR‐T amplification and an increased likelihood of experiencing Grade 3–4 CRS and ICANS.^28^ In 2017, the US FDA granted approval to the first‐ever CAR‐T product, CTL019, which specifically targets CD19 of R/R ALL, with a CR of up to 90%. However, the CR rate of CTL019 in R/R CLL, which also uses CD19 as a therapeutic target, was only 26%. Notably, Fraietta et al. compared CAR‐T cells from 41 patients in CTL019 remission and non‐remission groups to enhance the CR rate of CTL019 in R/R CLL. They found that the expansion of the two populations after CAR‐T reinfusion was significantly different. CAR T cells in the CR, subsequent disease recurrence and deterioration (PRTD), and partial response (PR) groups increased significantly within 2 weeks after infusion, whereas CAR‐T cells in the NR team expanded only slightly or did not increase. The CR group exhibited significantly higher persistence of CAR T cells compared to both the PR and NR groups, and the CR group maintained a higher level of CAR T cells from the peak to 6 months after transfusion.^96^

Elevated levels of circulating CAR‐T cells are frequently observed in conjunction with the initial response and sustained remission of hematologic tumors. These levels are not only linked to the peak of CAR‐T cell expansion in vivo but are also closely related to the duration of CAR‐T cells and entire expansion process. Understanding the level and duration of CAR‐T expansion is crucial for the initial prediction of the final effect.

### 4.5 CAR‐T cell targets

Many patients are prone to relapse after single‐target CAR‐T treatment, which can be divided into positive and negative relapses according to the tumor target antigen.^42^ Target antigen‐negative relapse is caused by a deletion or mutation of the target antigen locus, whereas target antigen‐positive relapse is caused by low efficacy or loss of CAR‐T cells. Multiple elements contribute to the limitation and reduced efficiency of CAR‐T cells, such as limited long‐term persistence, an immunosuppressive tumor microenvironment,^97^ and inherent T cell depletion‐related dysfunction.^77^, ^98^

Multi‐target CAR‐T may be an effective method for addressing relapse. Currently, The prevalent type of CAR‐T cells with multiple targets are dual‐target CAR‐T cells., which are mainly divided into bicistronic, tandem, sequential, and co‐transduced CAR‐T cells,^99^ CD19 combined with CD20 CAR‐T,^100^, ^101^ CD19 combined with CD22 CAR‐T,^102^ CD19 combined with CD20 BCMA CAR‐T,^102^, ^103^ and CD19, CD22 and CD20 triple‐target CAR‐T^104^, ^105^ showed that the infusion of multi‐target CAR‐T had better clinical outcomes than did single‐target CAR‐T with controllable CRS and ICANS.

## 5 TREATMENT PROCESS

### 5.1 Lymphodepletion regimen

Lymphodepletion before CAR‐T cell infusion may diminish the assault of the patient's immune system on CAR‐T cells by removing residual lymphocytes, removing immunosuppressive factors, and inducing costimulatory molecules. This establishes a “favorable” setting for the expansion and survival of CAR‐T cells in the body, thereby potentially enhancing the efficacy of CAR‐T therapy. Fludarabine and cyclophosphamide are commonly used as lymphodepletion regimens, usually performed 1 week prior to CAR‐T cell infusion.^106^ One study noted that fludarabine combined with cyclophosphamide produced better EFS than did cyclophosphamide alone.^40^ Furthermore, studies conducted on patients with B‐cell lymphomas^107^, ^108^, ^109^ and B‐ALL^110^, ^111^ showed enhanced treatment responses when lymphodepletion chemotherapy was administered before CAR T‐cell infusion. Lymphocyte‐depleting chemotherapy prior to CAR‐T cell infusion elevates serum IL‐15 levels, which, in turn, promote T cell proliferation. Thus, Kochenderfer et al. affirm that IL‐15 levels correspond with the peak expansion of CAR‐T cells in vivo.^107^ Finally, adequate lymphatic clearance is important for treatment efficacy and has the potential to mitigate CAR rejection, as evidenced by clinical trials in adult patients with B‐ALL and B‐NHL.

### 5.2 Allogeneic hematopoietic stem cell transplantation after CAR‐T infusion

CAR T cell therapy followed by consolidation allogeneic hematopoietic stem cell transplantation (allo‐HSCT) results in better LFS and OS than does CAR T cell therapy alone. Zhang et al. infused CD19 CAR‐T cells into 110 B‐ALL patients. The study reported a 1 year LFS rate of 58% and a 1 year OS rate of 64%. Among the patients, 73.5% underwent allo‐HSCT. Patients who underwent allo‐HSCT after receiving CAR T cells demonstrated significantly improved OS compared to those treated with CAR T cells alone (79.1% vs. 32.0%; *p* < 0.0001). Similarly, the LFS rate was also significantly higher in patients who received allo‐HSCT (76.9% vs. 11.6%; *p* < 0.0001) when compared to those who did not.^44^

A phase 1 trial that children and young adults aged 1 to 30 years who had R/R ALL or NHL were enrolled in a systematic manner. Notably, a 70% CR rate and a 60% negative MRD rate were found in B‐ALL. Moreover, 83% patients who achieved MRD negativity subsequently underwent allo‐HSCT following CAR‐T cell infusion. Importantly, at a median follow‐up time of 10 months, all patients remained free of disease recurrence.^78^ Therefore, CD19 CAR‐T cell therapy can effectively bridge allo‐HSCT in patients with CR or better MRD‐negative status.

### 5.3 Use of corticosteroids

Toxic reactions, such as CRS and ICANS, may occur in patients after CAR‐T treatment.^112^ CRS is a prevalent adverse event associated with CAR‐T therapy, characterized by the systemic release of large amounts of cytokines following the infusion of CAR‐T cells, leading to clinical manifestations including fever, hypoxia, hypotension, capillary leakage, and organ dysfunction. Conversely, ICANS manifests as neurotoxic symptoms, including confusion, psychosis, aphasia, and seizures, in patients following CAR‐T treatment. Corticosteroids are commonly used to manage severe toxic reactions linked to CAR T‐cell therapy. One study included 100 patients with R/R large B‐cell lymphoma; 9 (9%) patients developed severe CRS, 41 (41%) patients developed severe ICANS, 60 (60%) of whom used corticosteroids; the median cumulative dose of dexamethasone equivalence was 186 mg, with a range of 8–1803 mg, and the median treatment duration was 9 days, ranging from 1 to 30 days. Thus, higher cumulative doses of corticosteroids were found to be linked to noticeably reduced PFS. Moreover, higher cumulative doses of corticosteroids, along with their early and prolonged usage following CAR T‐cell infusion, were observed to be significantly associated with shorter OS.^113^ The findings indicate that it is advisable to administer corticosteroids at the minimal effective dose and for the shortest possible duration. Moreover, initiating their usage should be delayed whenever clinically viable during the management of CAR T‐cell therapy‐related toxicities.

## 6 STRATEGIES TO IMPROVE THE PERSISTENCE AND EFFICACY OF CAR‐T CELLS

### 6.1 Combined checkpoint blockade therapy

**FIGURE 2:**
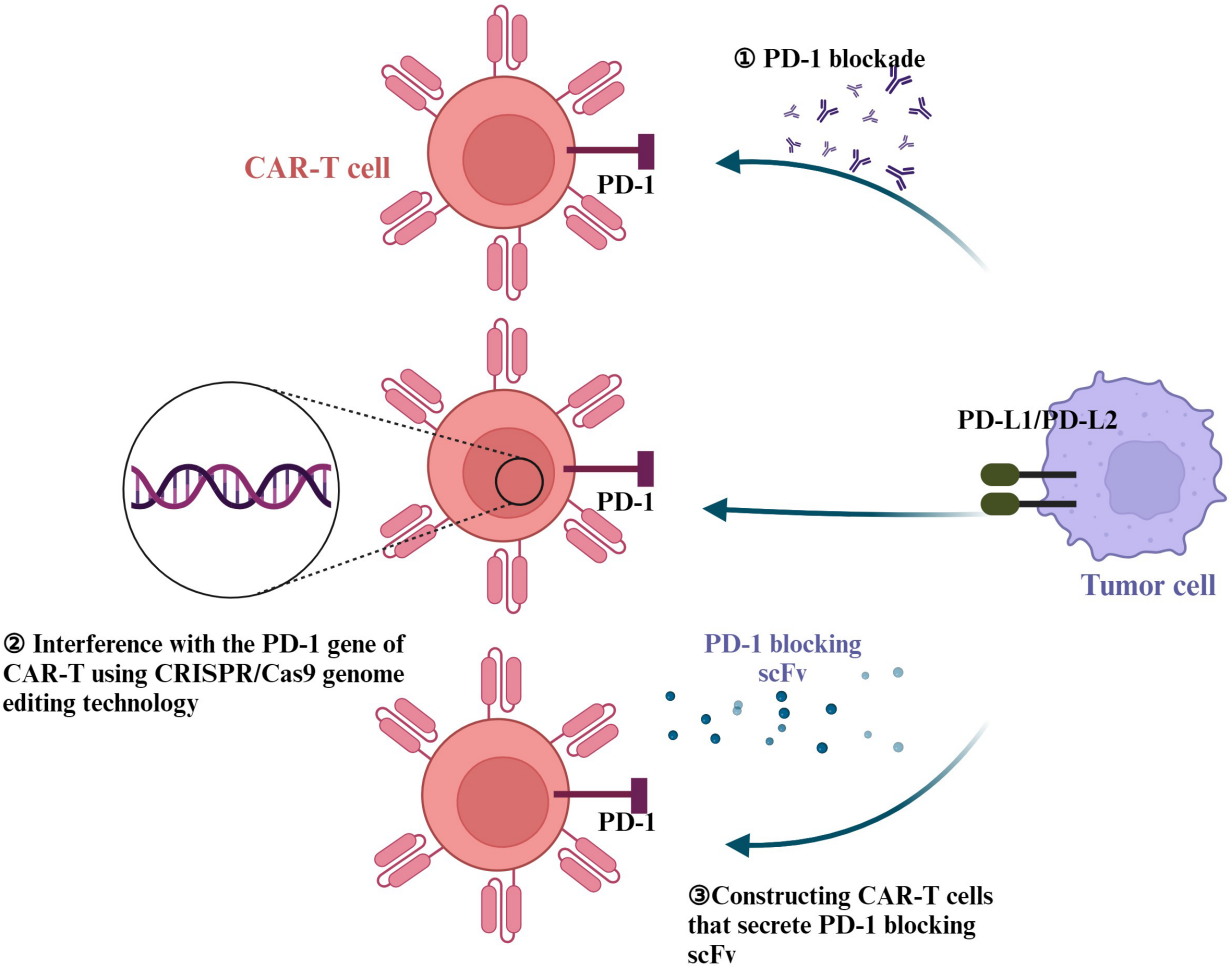
CAR‐T cells combine PD‐1 checkpoint blockade therapy in three ways.

Programmed cell death receptor 1 (PD‐1) is an immune checkpoint molecule^114^ expressed on various immune cells and plays an important role in immunosuppression. PD‐1 plays a significant role in T‐cell dysfunction by engaging with the programmed cell death ligand (PD‐L1), which is expressed on tumor cells. There are three approaches to generate CAR‐T cells: (i) Administering CAR‐T cell therapy followed by PD‐1/PD‐L1 inhibitors. (ii) Modifying the PD‐1 gene in CAR‐T cells utilizing CRISPR/Cas9 genome editing technology.^115^ (iii) Developing CAR‐T cells that release PD‐1 blocking scFv and concurrently employing CAR‐T cells with immune checkpoint blockade therapy, which is an effective method to enhance antitumor activity, the persistence of CAR‐T cells, and the generation of memory cell (Figure 2). Qian et al. developed a novel CAR‐T cell line that specifically targets CD19 and expresses the chimeric switch receptor PD‐1/CD28, called CD19‐PD‐1/CD28‐CAR‐T, and treated six patients with DLBCL with this CAR‐T cell line after CD19 CAR‐T cell therapy, which induced potent and durable remission.^116^ The research reveals that tumor cells in solid tumors highly express the inhibitory ligand PD‐L1, leading to a high depletion state of CAR‐T cells upon reinfusion. However, constructing a PD‐1 blockade structure within the CAR‐T cells could alleviate their depletion state post‐reinfusion, enhance their cytotoxic function, and extend patient survival.^117^ Furthermore, Lymphocyte‐activation gene 3 (LAG‐3), Cytotoxic T‐lymphocyte‐associated protein 4 (CTLA‐4) and T cell immunoglobulin and mucin‐domain containing‐3 (TIM‐3) are frequently encountered immune checkpoint molecules, and inhibition of these checkpoints can effectively improve CAR‐T cells' efficacy in the treatment of hematological disorders.

### 6.2 Manufacture of CAR‐T cells with a central memory or stem cell‐like memory phenotype

The initial T cell phenotype plays a crucial role in determining their subsequent clinical activity, and the selection of TCM and TSCM can promote sustained T cell proliferation and persistence. Xiuli Wang et al. employed CAR‐T immunotherapy for B‐NHL treatment following HSCT. They utilized in vivo expanded autologous TCM, which were transduced with lentivirus carrying CD19‐specific CARs to investigate CARs from various T‐cell subsets. The results of two feasibility studies, NHL1 and NHL2, were presented. In NHL1, T‐cell products derived from enriched CD8(+) TCM subpopulations were safely administered to eight patients expressing first‐generation CD19 CARs containing only the CD3ζ intracellular signaling domain. Conversely, in NHL2, a total of eight patients underwent safe administration of T‐cell products obtained from enriched CD4(+) and CD8(+) TCM subpopulations. These T‐cell products carried second‐generation CD19 CARs that included both the CD28 and CD3ζ internal domains. Notably, six of the eight patients (75%) remained 1 year PFS, and neither trial observed CRS or delayed hematopoietic engraftment.^118^ The data indicated that CD19 CAR TCM therapy after HSCT is safe and feasible.

Arcangeli et al., research indicates that CAR‐T cells derived from naive T cells (T_N_) and TSCM (CD62L+CD45RA+) and infused into mice demonstrate high expression of IFN‐γ, enhanced expansion capacity, and reduced exhaustion state. These CAR‐T_N/SCM_ cells could continuously suppress tumor cells and prevent relapse, while also showing lower incidences of CRS and ICANS.^119^ This suggests that various T cell subsets exhibit significant differences in proliferation capacity, self‐renewal ability, and long‐term survival. The lower the degree of T cell differentiation, the stronger their antitumor capability. CAR‐T_N/SCM_ has the potential to revolutionize the design of current CAR‐T cell therapies and, through its enhanced antitumor activity and persistence, can offer more effective and long‐lasting treatment for a broader range of patients. In the CRB‐402 study, BCMA CAR‐T cells (bb2121, Idecabtagene vicleucel) were co‐cultured with a PI3K inhibitor to form bb21217, leading to enrichment of memory‐like T cells in the CAR‐T cell population and reduction of highly differentiated and senescent T cells. The study results indicated that patients with higher proliferation levels and a lower degree of differentiation in memory‐like CAR‐T cells (CD27+CD28+) had a significantly longer duration of response (DOR) at the peak of expansion (27.2 months compared to 9.4 months).^120^ This suggests that co‐culturing with a PI3K inhibitor can optimize the phenotype of CAR‐T cells, rendering them more durably effective in patients and extending the duration of disease remission.

In addition to the factors (Table 1) that have been demonstrated to influence the effectiveness of CAR‐T cells in the above clinical and preclinical trials, many other factors are uncertain to have an effect, such as other genetic alterations, cytokine levels, and bacterial infections during the course of the disease; further experiments are needed to verify these results.

**TABLE 1:** Factors affecting CAR‐T efficacy in selected clinical trials.

Influencing Factors	NCT number	Targets	Malignancy type	Study and year of publication	Outcome	Adverse events
Malignancy type	NCT00924326 (phase1/2)	CD19	DLBCL/PMBCL (*n* = 28) CLL (*n* = 7) Low‐grade BCL (*n* = 8)	Cappell KM et al. 2020^23^	>3 year DOR:51% Among 48% for DLBCL/PMBCL 63% for low‐grade lymphoma 50% for CLL	Rare
Malignancy type	NCT03391466 (phase3)	CD19	LBCL (*n* = 359)	Locke FL et al. 2021^24^	24‐month EFS:41% versus 16% ORR:83% versus 50% 2‐years OS:61% versus 52%	≥3 grade adverse events:91% versus 83% ≥3 CRS:6% ≥3 ICANS:21%
Dose of CAR‐T	NCT01747486 (phase2)	CD19	CLL (*n* = 42)	Frey NV et al. 2020^121^	CR: 28% ORR: 44% The median OS:64 months	NA
Tumor burden	NCT01044069 (phase1)	CD19	B‐ALL (*n* = 53)	Park JH et al. 2018^28^	The median OS:12.9 months The median EFS:6.1 months CR:83%	CRS:26% Incidence of CRS and ICANS:a higher disease burden > a lower disease burden
Tumor burden	NCT02782351 (phase1)	CD19	Pediatric ALL (*n* = 24)	Wang S et al. 2021^122^	CR: 83.3% Low disease burden: 100% High disease burden: 50% NR:16.7%	1–2 CRS:83.3% 3 CRS:16.7% 1 ICANS:5% 3 ICANS:10%
TP53 mutation; HSCT	NCT03173417 (phase 1/2)	CD19	B‐ALL (*n* = 110)	Zhang X et al. 2020^44^	CR:93% 1 year LFS: 53% 1 year OS: 64% OS and LFS at 6 months: with TP53 mutation <without TP53 mutation	1–2 CRS:76% 3–4 CRS:16% 1 ICANS:7% 2–3 ICANS:14%
Costimulatory signal domain/transduction vector	NA	BCMA	MM (*n* = 640)	Roex G et al. 2020^80^	ORR: 80.5%; CR: 44.8% PFS: lentivirally transduced CAR‐T> retroviral transduced CAR‐T; 4‐1BB>CD28	CRS: 80.3% ICANS: 10.5%
The source of CAR‐T cells	NA	CD19	ALL (*n* = 953)	Anagnostou T et al. 2020^76^	CR:80% CR: autologous T‐cell origin (83%) >allogeneic T‐cell origin (55%)	3–4 CRS: 26% 3–4 ICANS: 12%
Chimeric antigen receptor (CAR) T cell levels	NCT02348216 (phase1)	CD19	B‐cell lymphoma (*n* = 111)	Neelapu SS et al. 2017^94^	ORR: 82% CR: 54% 18 months OS: 52%	3–4 CRS: 13% 3–4 ICANS: 28%
Chimeric antigen receptor (CAR) T cell levels	NCT01029366 (phase1)	CD19	CLL (*n* = 14)	Porter DL, et al. 2015^123^	ORR: 57% CR: 29% PR: 29%	CRS: 64%
Lymphodepletion, CAR‐T cell levels, IL‐15	NCT00924326 (phase 1/2)	CD19	DLBCL (*n* = 19) MCL (*n*‐1) FL (*n* = 2)	Kochenderfer JN, et al. 2017^107^	ORR: 73% CR: 55% PR: 18%	3–4 ICANS: 55%
The source of CAR‐T cells	NA	CD19	ALL (*n* = 953)	Anagnostou T, et al.2020^76^	CR: 80% CR: autologous T‐cell origin(83%)>allogeneic T‐cell origin(55%)	3–4 CRS: 26% 3–4 ICANS: 12%
Corticosteroids	NA	CD19	LBCL (*n* = 100)	Strati P, et al. 2021^113^	CR:57% PFS:8 months	3–4 CRS: 9% 3–4 ICANS: 41%

Abbreviations: CR, complete remission; CRS, cytokine release syndrome; DOR, duration of response; EFS, event‐free survival; ICANS, immune cell‐associated neurological syndrome; LFS, leukemia free survival; NR, no response; ORR, overall response; OS, overall survival.

## 7 CONCLUSIONS

Identifying factors that contribute to determining the efficacy of CAR‐T cell therapy for hematologic malignancies is crucial. These factors include tumor characteristics, the tumor microenvironment, and patients' immune function, as well as the structure, construction method, and in vivo expansion values of CAR‐T cells. Additionally, lymphodepletion chemotherapy and previous treatments are pertinent. These elements offer avenues for predicting and enhancing the efficacy of CAR‐T cells post‐reinfusion in the subsequent pre‐infusion phase of CAR‐T therapy for hematologic tumors. Moreover, they aid in ascertaining the optimal characteristics for forecasting the most favorable treatment outcome. Thus, treatment can be meticulously tailored to the individual, based on both patient and CAR‐T cell characteristics, to maximize efficacy.

Therefore, tailoring treatment plans to the specific characteristics of the patient and the CAR‐T cells is crucial to maximizing efficacy. Future research directions should include investigating the molecular and genetic differences across various malignancies and formulating strategies to reduce tumor burden; monitoring and regulating cytokines in the tumor microenvironment to improve CAR‐T cell activity and survival; innovating new costimulatory domains and gene delivery methods to enhance efficacy and safety; optimizing lymphodepletion regimens to improve CAR‐T cell expansion and minimize side effects; and employing immune profiling and immunomodulatory therapies to predict and enhance treatment outcomes. By addressing these issues, the efficacy and safety of CAR‐T cell therapy could be significantly improved, thus providing better treatment outcomes for patients with hematologic malignancies.

## AUTHOR CONTRIBUTIONS

**Delian Zhou:** Writing – original draft (equal). **Xiaojian Zhu:** Writing – review and editing (equal). **Yi Xiao:** Writing – review and editing (equal).

## FUNDING INFORMATION

This research was funded by National Natural Science Foundation of China, grant number 82070213.

## CONFLICT OF INTEREST STATEMENT

The authors declare no conflict of interest.

## Data Availability

Data sharing is not applicable to this article as no new data were created or analyzed in this study.
